# Relationship between non–high-density lipoprotein cholesterol and carotid atherosclerosis in normotensive and euglycemic Chinese middle-aged and elderly adults

**DOI:** 10.1186/s12944-017-0451-4

**Published:** 2017-03-16

**Authors:** Hui Ma, Huandong Lin, Yu Hu, Xiaoming Li, Wanyuan He, Xuejuan Jin, Jian Gao, Naiqing Zhao, Boshen Pan, Xin Gao

**Affiliations:** 1 Department of Geriatrics, Zhong Shan Hospital, Fudan University, Shanghai, 200032 China; 2 Department of Endocrinology and Metabolism, Zhong Shan Hospital, Fudan University, Shanghai, 200032 China; 3 Department of Ultrasonography, Zhongshan Hospital, Fudan University, Shanghai, 200032 China; 4 Clinical Epidemiology Center, Zhong Shan Hospital, Fudan University, Shanghai, 200032 China; 5 Department of Clinical Nutrition, Zhong Shan Hospital, Fudan University, Shanghai, 200032 China; 6 Department of Biostatistics, College of Public Health, Fudan University, Shanghai, 200032 China; 7 Department of Laboratory Medicine, Zhongshan Hospital, Fudan University, Shanghai, 20032 China

**Keywords:** Non-HDL-C, Carotid intima-media thickness (CIMT), Carotid plaque, Carotid atherosclerosis

## Abstract

**Background:**

We investigate whether non–high-density lipoprotein cholesterol (non-HDL-C) provides a better estimate of cardiovascular risk than other lipid profiles in normotensive and euglycemic middle-aged and elderly adults.

**Methods:**

A total of 512 males and 958 females were enrolled from the Changfeng Study. A standard interview, anthropometric measurements and laboratory analyses were performed for each participant. Bilateral carotid intima-media thicknesses (CIMTs) were measured using ultrasonography, and the presence of carotid plaques was assessed.

**Results:**

The mean values of non-HDL-C were 3.4 ± 0.8 mmol/l and 3.6 ± 0.9 mmol/l for male and female subjects, respectively. Compared with female subjects with non-HDL-C in the first quartile, female subjects with non-HDL-C in the fourth quartile had 1.317-fold increased risks for carotid plaques after adjusting for conventional cardiovascular disease (CVD) risk factors and increasing quartiles of all lipid levels. Non-HDL-C was positively associated with the CIMT after adjusting for CVD risk factors in female subjects (β = 0.062, *P* = 0.034).

**Conclusions:**

These results suggest that non-HDL-C is independently associated with carotid atherosclerosis in normotensive and euglycemic females.

## Background

Lipoprotein abnormalities play a role in the accelerated atherosclerosis. Although low-density lipoprotein cholesterol (LDL-C) has been the primary measure used to estimate cardiovascular disease (CVD) risk by guidelines for over 3 decades, there are now many studies demonstrating consistent outperformance by non–high-density lipoprotein cholesterol (non-HDL-C) [1, 2]. Moreover, recommendations from the International Atherosclerosis Society Expert Dyslipidemia Panel [3] and the National Lipid Association [4] recommend non-HDL-C also as a primary target of therapy. Non-HDL-C, which represents the total cholesterol content of apolipoprotein B (apo-B) containing lipoproteins, is particularly useful in risk assessment for diabetic patients rather than LDL-C levels [5]. Non–HDL-C is a quick and simple calculation of total cholesterol (TC) minus high-density lipoprotein cholesterol (HDL-C), and can be obtained in the non-fasting state without affecting results. In studies, researchers have shown that non-HDL-C is a better marker of coronary heart disease (CHD) risk than LDL-C in patients with CVD and diabetes [6, 7]. In the Strong Heart Study, Lu et al. [8] found non-HDL-C to be a better predictor of cardiovascular events in patients with diabetes compared to LDL-C [hazard ratio (HR) 2.23 and 1.80 for the highest tertile of non-HDL-C among men and women, respectively]. In diabetic men of the Health Professionals’ Follow-up Study, comparisons of nested models highlighted that non-HDL-C add significantly to the prediction of CVD risk beyond LDL-C [9]. Tohidi et al demonstrated that among the non-diabetic population, non-HDL-C was a significant predictor of incident CVD in both genders; similar to the association shown among non-diabetic Arab community [10]. However, the Rancho Bernardo Study of 1386 women and 1094 men (mean age, 69 years) found that non-HDL-C was not superior to individual lipids, lipoproteins, or their ratios in the prediction of fatal CHD and CVD in a 10 year follow-up [11]. As shown by previous reports, non-HDL-C may be associated with the prognostic factors of CVD, such as diabetes [12] and hypertension [13], which may themselves affect CVD events. Therefore, the relationship between non-HDL-C and atherosclerosis may be confounded by the presence of hyperglycemia and hypertension. In addition, it has been shown that non-HDL-C values vary between different ethnicities. The above large scale studies were mostly confined to the individuals in Europe and North America, which could not reflect the features of other ethnicities. Few studies have directly compared the relative usefulness of conventional lipid particles for prediction of subclinical atherosclerosis in the Chinese population. Given the risk for CVD in Chinese middle-aged and elderly adults [14], more evidence is needed to evaluate the association between non-HDL-C and atherosclerosis.

Accordingly, in this study we aim to compare the association of non-HDL-C, LDL-C, HDL-C and TC with carotid intima-media thickness (CIMT) and carotid plaque, reliable markers of the severity of atherosclerosis [15] in Chinese middle-aged and elderly adults with normal blood pressure and normal glucose tolerance (NGT).

## Methods

### Study population

The subjects were participants in the Changfeng Study, a community-based study of chronic diseases among middle-aged and elderly individuals which has been described elsewhere [16, 17]. From June 2009 to June 2012, 5485 participants were initially enrolled. We excluded 4015 participants for the following reasons: lack of physical examination and laboratory assessments (*n* = 110), prevalent CVD (myocardial infarction, stroke, or) (*n* = 404), prevalent hemodialysis (*n* = 2), prevalent hypertension (systolic blood pressure ≥ 140 mmHg, diastolic blood pressure ≥90 mmHg, the use of antihypertensive medications, or diagnosed hypertension) (*n* = 2672), prevalent diabetes or pre-diabetes (fasting glucose ≥5.6 mmol/L, OGTT (oral glucose tolerance test) 2 h glucose ≥7.8 mmol/L, the use of hypoglycemic medication, or diagnosed diabetes) (*n* = 812), triglycerides ≥4.5 mmol/L or the use of lipid-lowering therapy (*n* = 12), and use of the antiplatelet agents (*n* = 3). Finally, 1470 subjects (512 males and 958 females) were included in the analysis.

### Ethics statement

The study was approved by the ethical committee of Zhongshan Hospital, Fudan University and was conducted in accordance with the guidelines of the Declaration of Helsinki. Written informed consent was obtained from all participants.

### Measurements

Interviews, physical examinations and ultrasound scans were performed at the Changfeng Community Health Service Center. Trained nurses interviewed the participants and obtained their medical history and lifestyle. Weight and height were measured while the participant was clothed in a light gown. The waist circumference was measured midway between the lowest rib margin and the iliac crest in a standing position. The hip circumference was measured at the widest level over the greater trochanters. The waist-to-hip ratio (WHR) was calculated from these values. The body mass index (BMI) was calculated as the weight divided by the height squared (kg/m^2^). The resting blood pressure was measured 3 times, and the mean value was used for the analysis. Blood samples were obtained after a fasting period of at least 10 h. TC, HDL-C and triglycerides (TG) were measured using a model 7600 automated bio-analyser (Hitachi, Tokyo, Japan). The level of LDL-C was calculated using the Friedewald equation. Non-HDL-C is calculated as TC minus HDL-C [7]. The fasting blood glucose (FBG) and 2 h glucose levels following a 75 g oral glucose challenge (PPG) (postprandial blood glucose) for non-diabetics were measured using the glucose oxidase method. Serum insulin was measured using electrochemiluminescence immunoassay using an immunoassay analyzer (Roche Cobas-6001, Switzerland) (coefficient of variation <5.0%). Homeostasis model assessment index for insulin resistance (HOMA-IR) and beta cell function (HOMA-B%) were used to estimate insulin sensitivity and insulin secretion [18].

Carotid ultrasonography was performed (using a GE Logic P5 (GE Healthcare, Milwaukee, USA) scanner with a 10-MHz probe) on all participants by an experienced radiologist who was blinded to the participants’ details. Right and left CIMT were measured in the common carotid artery approximately 1 cm proximal to the bifurcation at the far wall during end diastole. The CIMT was quantified at plaque-free sections of the carotid arteries as the distance between the lumen-intima and media-adventitia interfaces. Three values were measured on each side, and the average CIMT was used for the analysis. Carotid plaque was defined as the presence of focal wall thickening resulting in a thickness that is ≥ 50% greater than that of the surrounding vessel wall or as a focal region with a CIMT greater than 1.5 mm that protrudes into the lumen that is distinct from the adjacent boundary, according to American Society of Echocardiography [19]. The near and far walls of bilateral common carotid arteries, the carotid bifurcation, and the internal carotid artery were scanned for the presence of plaques. Repeated measurements on the same subjects (performed in 103 subjects) yielded an intraclass correlation coefficient of 0.93 (95% confidence interval, 0.91 to 0.96).

Hypertension was defined according to the Seventh Report of the Joint National Committee [20]. Glucose tolerance was evaluated based on OGTT as set by the American Diabetes Association 2010 criteria [21]. The diagnosis of dyslipidaemia was based on Chinese guidelines for the prevention and treatment of dyslipidaemia in adults [22]. The diagnosis of CVD and peripheral vascular disease (PAD) was based on self-reports and confirmed using medical records.

### Statistical analyses

The data were expressed as the means ± SD, frequencies or medians with 25th and 75th percentiles. Skewed variables were logarithmically transformed to improve normality prior to analysis. Association between increasing quartile of lipid values and carotid atherosclerosis was initially assessed with a chi-square analysis, stratified by gender. Linear regression analysis was performed to examine the association between the lipid profiles and the CIMT. Logistic regression was then employed to further quantify the association between lipid level quartiles and carotid plaque, with the lowest quartile as reference. The multivariate regression analysis was adjusted, as follows: model 1: age, SBP, DBP, FBG, PPG, BMI, WHR, smoking (yes or no), HOMA-IR and HOMA-B%. In model 2, we adjusted for all lipid variables in addition to risk factors in model 1 to assess the independent association of each lipid profile with carotid atherosclerosis. Odds ratios (ORs) were calculated for a 1-unit increase in the lipid profiles. SPSS 16.0 for Windows (SPSS 16.0 Inc, USA) was used to perform the statistical analyses. All statistical tests were two tailed, and p-values < 0.05 were considered significant.

## Results

### Population characteristics

A total of 512 males and 958 females were evaluated. The demographic and clinical characteristics of the study subjects are shown in Table 1. The mean values of CIMT were 0.746 ± 0.142 mm and 0.694 ± 0.119 mm in males and females, respectively. The prevalence of carotid plaques was 27.0% and 14.4%. The mean values of non-HDL-C were 3.4 ± 0.8 mmol/l and 3.6 ± 0.9 mmol/l. A total of 21.6% of the subjects were current smokers. The differences between genders in terms of age, BMI, WHR, SBP, DBP, TC, LDL-C, HDL-C, non-HDL-C, smoking, HOMA-IR, HOMA-B%, CIMT, and the presence of carotid plaques were statistically significant.

**Table 1:** Characteristics of the study subjects

Variables	All	Male subjects	Female subjects	P value between genders
	*n* = 1470	*n* = 512	*n* = 958	
Age (ys)	57.7 (8.1)	59.0 (8.3)	57.0 (8.0)	<0.001
BMI (kg/m^2^)	22.9 (2.9)	23.1 (3.0)	22.7 (2.9)	0.029
WHR	0.868 (0.069)	0.901 (0.069)	0.850 (0.061)	<0.001
Current smoker, n (%)	317 (21.6%)	296 (57.8%)	21 (2.2)	<0.001
SBP (mmHg)	120.4 (11.1)	121.8 (11.2)	119.6 (11.0)	<0.001
DBP (mmHg)	71.5 (7.7)	73.1 (7.8)	70.7 (7.5)	<0.001
TC (mmol/L)	5.0 (0.9)	4.8 (0.9)	5.2 (0.9)	<0.001
LDL cholesterol (mmol/L)	2.9 (0.8)	2.8 (0.7)	3.0 (0.8)	<0.001
HDL cholesterol (mmol/L)	1.5 (0.4)	1.4 (0.4)	1.6 (0.4)	<0.001
Non-HDL-C (mmol/L)	3.5 (0.9)	3.4 (0.8)	3.6 (0.9)	<0.001
TG (mmol/L)	1.3 (0.6)	1.4 (0.7)	1.3 (0.6)	0.167
FBG (mmol/L)	4.9 (0.4)	4.9 (0.4)	4.9 (0.3)	0.608
PPG (mmol/L)	5.6 (1.1)	5.6 (1.2)	5.6 (1.1)	0.391
HOMA-IR	1.4 (0.9–1.9)	1.2 (0.8–1.8)	1.4 (1.0–1.9)	<0.001
HOMA-B%	93.3 (67.0–126.2)	83.9 (58.2–123.4)	97.3 (71.7–128.9)	<0.001
CIMT (mm)	0.712 (0.130)	0.746 (0.142)	0.694 (0.119)	<0.001
Carotid plaque (n,%)	276 (18.8%)	138 (27.0%)	138 (14.4%)	<0.001

Data are mean (SD) or percentage of subjects or median (interquartile range)

*BMI* body mass index, *WHR* waist-hip-ratio, *FBG* fasting blood glucose, *PPG* OGTT 2 h blood glucose, *SBP* systolic blood pressure, *DBP* diastolic blood pressure, *TC* total cholesterol, *TG* triglyceride, *HDL*-*C* high density lipoprotein cholesterol, *LDL*-*C* low density lipoprotein cholesterol, *Non*-*HDL*-*C* non high density lipoprotein cholesterol, *HOMA*-*IR* homeostasis model assessment index for insulin resistance, *HOMA*-*B* homeostasis model assessment index for beta cell function

The mean lipid values were well within the recommended range by the guidelines of Joint Committee for Developing Chinese guidelines on Prevention and Treatment of Dyslipidemia in Adults [22]. The majority of the study population (*n* = 1082, 73.6%) had LDL-C levels <3.37 mmol/l, whereas 36.6% (*n* = 538) had LDL-C <2.6 mmol/l. Only 1.4% (*n* = 20) of participants had LDL-C ≥ 4.9 mmol/l. Low HDL-C (<1.04 mmol/l was observed in 113 cases (7.7%) of the study population.

### Association of carotid plaque and lipid levels

**Fig. 1:**
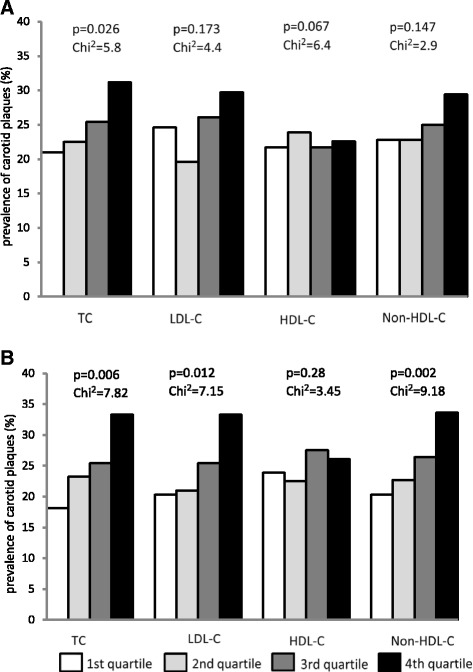
**a** Prevalence of carotid plaque across quartile of lipid levels in male subjects. TC: 2.65–4.15mmol/L 1st quartile; 4.17–4.69mmol/L 2nd quartile; 4.71–5.28mmol/L 3rd quartile; 5.29–8.84mmol/L 4th quartile. LDL-C: 1.05–2.27mmol/L 1st quartile; 2.28–2.69mmol/L 2nd quartile; 2.7–3.23mmol/L 3rd quartile; 3.24–6.65mmol/L 4th quartile. HDL-C: 0.21–1.12mmol/L 1st quartile; 1.13–1.3mmol/L 2nd quartile; 1.31–1.55mmol/L 3rd quartile; 1.56–4mmol/L 4th quartile. Non-HDL-C: 1.51–2.78mmol/L 1st quartile; 2.79–3.3mmol/L 2nd quartile; 3.31–3.94mmol/L 3rd quartile; 3.96–7.87mmol/L 4th quartile. **b** Prevalence of carotid plaque across quartile of lipid levels in female subjects. TC: 3.07–4.62mmol/L 1st quartile; 4.63–5.15mmol/L 2nd quartile; 5.16–5.73mmol/L 3rd quartile; 5.74–9.62mmol/L 4th quartile. LDL-C: 1.12–2.45mmol/L 1st quartile; 2.46–2.94mmol/L 2nd quartile; 2.95–3.47mmol/L 3rd quartile; 3.48–6.81mmol/L 4th quartile. HDL-C: 0.5–1.33mmol/L 1st quartile; 1.34–1.57mmol/L 2nd quartile; 1.58–1.85mmol/L 3rd quartile; 1.86–3.27mmol/L 4th quartile. Non-HDL-C: 1.45–2.98mmol/L 1st quartile; 2.99–3.56mmol/L 2nd quartile; 3.57–4.17mmol/L 3rd quartile; 4.18–8mmol/L 4th quartile

Figure 1 shows the prevalence of carotid plaque according to increasing quartiles of lipid levels. The presence of carotid plaque increased significantly across increasing quartiles of TC level in the male subjects, whereas prevalence increased significantly across increasing quartiles of TC, LDL-C and non-HDL-C levels in the female subjects. The highest chi-square value was observed for the relationship of non-HDL-C levels in the prevalence of carotid plaque (chi-square = 9.18) in females.

In a multivariate model adjusting for age, BMI, WHR, FBG, PPG, SBP, DBP, smoking, logHOMA-IR and logHOMA-B%, male subjects with TC and non-HDL-C in the fourth quartiles had 2.213-fold and 2.149-fold increased risks for carotid plaques relative to those in the lowest quartiles (Table 2, model 1). In the meanwhile, female subjects with TC and non-HDL-C in the fourth quartiles had 1.786-fold and 1.590-fold increased risks (Table 2, model 1). After simultaneously controlling for increasing quartiles of all lipid levels (Table 2, model 2), only females with non-HDL-C in the fourth quartiles had 1.317-fold increased risks for carotid plaques relative to those in the lowest quartiles. On the other hand, no such association was seen with TC, LDL-C and HDL-C.

**Table 2:** Adjusted odd ratios for carotid plaque according to lipid levels in male and female subjects

	Male subjects				
	1	2	3	4	Trend across quartiles
TC					
Model 1	1 (ref)	1.263 (0.680–2.374)	1.523 (0.827–2.807)	2.213 (1.199–4.084)	0.071
Model 2	1 (ref)	1.209 (0.550–2.502)	1.550 (0.543–4.422)	2.129 (0.507–8.944)	0.765
LDL-C					
Model 1	1 (ref)	0.757 (0.408–1.402)	1.137 (0.627–2.062)	1.498 (0.827–2.711)	0.176
Model 2	1 (ref)	0.511 (0.253–1.030)	0.482 (0.182–1.278)	0.428 (0.117–1.560)	0.295
HDL-C					
Model 1	1 (ref)	0.787 (0.427–1.451)	0.837 (0.444–1.581)	0.746 (0.305–1.197)	0.206
Model 2	1 (ref)	0.767 (0.408–1.443)	0.742 (0.369–1.490)	0.776 (0.326–1.183)	0.289
Non-HDL-C					
Model 1	1 (ref)	1.178 (0.628–2.209)	1.302 (0.712–2.422)	2.149 (1.121–4.118)	0.069
Model 2	1 (ref)	1.322 (0.624–2.801)	1.606 (0.525–4.910)	2.657 (0.562–6.566)	0.209
	Female subjects				
	1	2	3	4	Trend across quartiles
TC					
Model 1	1 (ref)	1.141 (0.645–2.017)	1.301 (0.745–2.273)	1.786 (1.044–3.056)	0.142
Model 2	1 (ref)	1.149 (0.572–2.306)	1.312 (0.504–3.412)	1.795 (0.490–5.575)	0.787
LDL-C					
Model 1	1 (ref)	0.915 (0.518–1.614)	1.165 (0.677–2.003)	1.615 (0.957–2.725)	0.128
Model 2	1 (ref)	0.834 (0.431–1.613)	1.015 (0.420–2.326)	1.209 (0.368–3.967)	0.735
HDL-C					
Model 1	1 (ref)	0.866 (0.509–1.474)	1.184 (0.695–2.018)	0.987 (0.561–1.737)	0.716
Model 2	1 (ref)	0.776 (0.446–1.348)	1.051 (0.580–1.903)	0.878 (0.376–1.609)	0.616
Non-HDL-C					
Model 1	1 (ref)	0.951 (0.539–1.679)	1.230 (0.853–1.957)	1.590 (1.034–2.708)	0.035
Model 2	1 (ref)	0.724 (0.457–1.469)	1.064 (0.942–1.776)	1.317 (1.019–2.511)	0.074

Model 1 adjusted for age, BMI, WHR, FBG, PPG, SBP, DBP, smoking, logHOMA-IR and logHOMA-B%, Model 2 adjusted for increasing quartiles of all the other lipid levels in addition to the risk factors adjusted in Model 1

*BMI* body mass index, *WHR* waist-hip-ratio, *FBG* fasting blood glucose, *PPG* OGTT 2 h blood glucose, *SBP* systolic blood pressure, *DBP* diastolic blood pressure, *TC* total cholesterol, *HDL*-*C* high density lipoprotein cholesterol, *LDL*-*C* low density lipoprotein cholesterol, *Non*-*HDL*-*C* non high density lipoprotein cholesterol, *HOMA*-*IR* homeostasis model assessment index for insulin resistance, *HOMA*-*B* homeostasis model assessment index for beta cell function

### Association of CIMT and non-HDL-C levels

The multiple linear regression revealed that the non-HDL-C was positively correlated with the CIMT after adjusting for traditional CVD risk factors including age, BMI, WHR, FBG, PPG, SBP, DBP, TC, LDL-C, HDL-C, smoking, logHOMA-IR and logHOMA-B% in female subjects (Table 3). However, no such association existed in male subjects (Table 3).

**Table 3:** Association of Non-HDL-C with CIMT by linear regression analysis

	Male subjects				Female subjects			
	β	Standard β	95% CI	*P*	β	Standard β	95% CI	*P*
Unadjusted	0.008	0.050	0.006–0.023	0.263	0.016	0.114	0.007–0.024	<0.001
Model 1	0.004	0.031	0.003–0.021	0.319	0.011	0.075	0.004–0.020	0.010
Model 2	0.002	0.013	0.001–0.117	0.651	0.009	0.062	0.002–0.017	0.034

Dependent variable: CIMT; Independent variable: Non-HDL-C

β: regression coefficient. β was calculated for a 1-unit increase in Non-HDL-C

Model 1 adjusted for age, BMI, WHR, FBG, PPG, SBP, DBP, smoking, logHOMA-IR and logHOMA-B%, Model 2 adjusted for all the other lipid levels in addition to the risk factors adjusted in Model 1

*BMI* body mass index, *WHR* waist-hip-ratio, *FBG* fasting blood glucose, *PPG* OGTT 2 h blood glucose, *SBP* systolic blood pressure, *DBP* diastolic blood pressure, *TC* total cholesterol, *HDL*-*C* high density lipoprotein cholesterol, *LDL*-*C* low density lipoprotein cholesterol, *Non*-*HDL*-*C* non high density lipoprotein cholesterol, *HOMA*-*IR* homeostasis model assessment index for insulin resistance, *HOMA*-*B* homeostasis model assessment index for beta cell function, *CIMT* carotid intima-media thickness

## Discussion

Our study showed that non-HDL-C was independently associated with carotid atherosclerosis in a normotensive and euglycemic female population. The CIMT and the prevalence of carotid plaques significantly increased with increasing non-HDL-C after adjusting for conventional CVD risk factors and all lipid levels in female subjects. However, no relationship was found between non-HDL-C and carotid atherosclerosis in male subjects.

Non-HDL-C has recently become a more and more important topic in cardiovascular research [1–3]. Non-HDL-C reflects the number of atherogenic particles present in the plasma. This includes very low density lipoproteins (VLDL), intermediate density lipoproteins (IDL) and LDL. Although a number of studies have demonstrated that non-HDL-C activity is associated with the development of cardiovascular events [1–5], it is still debated whether non-HDL-C provides a better estimate of cardiovascular risk than other lipid profiles. The participants in the Strong Heart Study [8] and the Health Professionals’ Follow-up Study [9] were diabetic patients. Although Tohidi’s study [10] was conducted using nondiabetic participants, these studies excluded only subjects with fasting blood glucose levels ≥7.0 mmol/L, 2 h plasma glucose ≥ 11.1 mmol/L, or subjects who were using hypoglycaemic medications. Therefore, the results may be confounded by the inclusion of subjects with impaired glucose regulation or postprandial hyperglycemia. Moreover, the previous studies enrolled study populations with different proportions of hypertension. As noted, recent studies have demonstrated that there was a close association between non-HDL-C and cardiovascular risk factors, such as hyperglycemia and hypertension [12, 13]. Therefore, the relationship between carotid atherosclerosis and non-HDL-C would be affected by the chronic effects of CVD risk factors. The impact of residual confounding factors would remain after adjusting for CVD risk factors in previous studies. To exterminate the various effects of CVD risk factors on the relationship between carotid atherosclerosis and non-HDL-C, we explored the relationship in the subjects with normal blood pressure and NGT. Thus, the association between non-HDL-C and carotid atherosclerosis was not confounded.

Non-HDL-C is the sum of the masses of cholesterol in the atherogenic apoB lipoprotein particles. In of principle, non–HDL-C represents a broader, more inclusive measure of atherogenic risk. Atherogenic dyslipidemia consists of the combination of an increase in VLDL, with a reduction of levels of HDL-C, also accompanied by a high proportion of small and dense LDL particles [4]. Atherogenic dyslipidemia is considered the main cause of the residual risk of experiencing cardiovascular disease (CVD), which is still presented by any patient on treatment with statins despite maintaining LDL-C levels below the values considered to be the objective [23]. The finding suggests opportunities for further risk reduction of CVD. Emerging research has identified potential surrogate lipid markers for assessing cardiovascular risk, including non–HDL-C. The dynamic flux of lipoproteins between subtypes under direction of lipoprotein lipase (LPL) and cholesterol ester transfer protein (CETP) makes direct assessment of total atherogenic burden a challenge, which is significantly improved by non–HDL-C [24, 25]. Several years ago, non-HDL-C was highlighted as an important secondary lipid therapeutic goal in the U.S. National Cholesterol Education Program’s Adult Treatment Panel [26]. The recent recommendations from the International Atherosclerosis Society Expert Dyslipidemia Panel [3] and the National Lipid Association [4] also recommend non–HDL-C as a primary target of therapy.

Using non-HDL-C levels offers several practical advantages over other traditional lipid parameters for routine clinical practice. First, measurement of the non–HDL-C level has no additional cost or inconvenience because it is easily calculated from the standard lipid profile without the need for prior fasting. Second, non-HDL-C level might also identify a group of individuals who have all of the potentially atherogenic lipid fractions [LDL-C, lipoprotein (a) [11], IDL-C [5] and VLDL-C remnants [5]. Finally, they can also be an exact marker in diabetic patients with hypertriglyceridemia, which can cause LDL-C level calculations using the Friedewald formula to be potentially inaccurate [3, 4]. In addition, the adoption of non–HDL-C across all levels of triglycerides would substantially simplify implementation of clinical guidelines.

In the present study, we did not find the association between non-HDL-C level and carotid atherosclerosis in male as the previous studies reported [27, 28]. Our study showed that non-HDL-C levels are better targets compared with other lipid profiles in female subjects, which presented as a stronger linear relationship with increased CIMT and a more powerful discriminatory ability for carotid plaque prediction. The results were in line with another population-based study in America [29]. Mora et al. found among initially healthy women, those with LDL-C < 121 mg/dl (3.13 mmol/l), non-HDL-C was more closely associated with coronary risk than LDL-C [29]. The diversity may be due to the sex-related differences and ethnic differences in lipid level and atherosclerosis [30]. Additionally, the non-HDL-C levels of the male subjects in this study were relatively lower in comparison with females, while ages, BMI, WHR, blood pressure, and current smokers were higher, which may contribute to the insignificant correlation between non-HDL-C and carotid atherosclerosis in males. However, we cannot further elaborate on the reasons behind this phenomenon.

Thus, our results may have important clinical implications with adding non-HDL-C into the evaluation tools for improving the detection of CVD among community based female subjects. The American College of Cardiology [6], American Diabetes Association [31] and National Lipid Association [25] have already recommended reporting non-HDL-C levels as part of routine lipid panel results. In the context of patient care, screening based on the non-HDL-C would enable clinicians to provide early interventions. An early evaluation of the non-HDL-C would be advantageous for the early detection of CVD, and individuals with increased non-HDL-C might benefit from more aggressive lifestyle modifications and dietary regimen.

Our study has limitations. It was a cross-sectional study, so no conclusion about cause and effect can be made. Our results reflect the relationship within the middle-aged and elderly subjects, limiting our external validity. Indeed, using the Friedwald formula there in an inherent risk of miscalculating the actual LDL-C levels, especially in the presence of elevated TC. In our cohort there were no patients with TC > 10.36 mmolL (400 mg/dl). The strengths of the study were that it was conducted on a population-based cohort. Additionally, we used both CIMT and carotid plaques as the surrogate markers of atherosclerosis [32].

## Conclusions

In conclusion, our study shows that among the traditional lipid measures, non-HDL-C was more strongly associated with subclinical atherosclerosis as estimated by measurement of CIMT and carotid plaque in community based female subjects. The third Adult Treatment Panel of the NCEP has recommended LDL-C as the primary target of therapy and the use of non–HDL-C as a secondary target of lipid lowering for individuals with TG concentrations ≥2.26 mmol/L (200 mg/dL) [33]. However, many individuals are at increased risk of CHD due to elevated concentrations of atherogenic lipoproteins not reflected in LDL-C measurement. Our results support the viewpoint that non–HDL-C may be a potentially better marker in evaluating the cardiovascular risk than LDL-C. The use of non-HDL-C instead of LDL-C as a primary target for cholesterol lowering therapy deserves further investigation.

## Abbreviations

apo-B: Apolipoprotein B
BMI: Body mass index
CETP: Cholesterol ester transfer protein
CHD: Coronary heart disease
CIMT: Carotid intima-media thickness
CVD: Cardiovascular disease
FBG: Fasting blood glucose
HDL-C: High-density lipoprotein cholesterol
HOMA-B%: Homeostasis model assessment index for beta cell function
HOMA-IR: Homeostasis model assessment index for insulin resistance
HR: Hazard ratio
IDL: Intermediate density lipoproteins
LDL-C: Low-density lipoprotein cholesterol
LPL: Lipoprotein lipase
NGT: Normal glucose tolerance
non-HDL-C: Non–high-density lipoprotein cholesterol
OGTT: Oral glucose tolerance test
Ors: Odds ratios
PPG: Postprandial blood glucose
TC: Total cholesterol
TG: Triglyceride
VLDL: Very low density lipoproteins
WHR: Waist-to-hip ratio
